# Supplementary material for: Splice-Junction-Based Mapping of Alternative Isoforms in the Human Proteome
Source: Cell Rep. Author manuscript; Available in PMC 2020 Jan 15. (PMC6961840; doi:10.1016/j.celrep.2019.11.026)
Supplement: 2 [file NIHMS1546469-supplement-2.zip › DF1/PXD010154/Lung/Lung_40_LMF2_PAAGQGARCGAGLGGVEWAGVER.pdf]

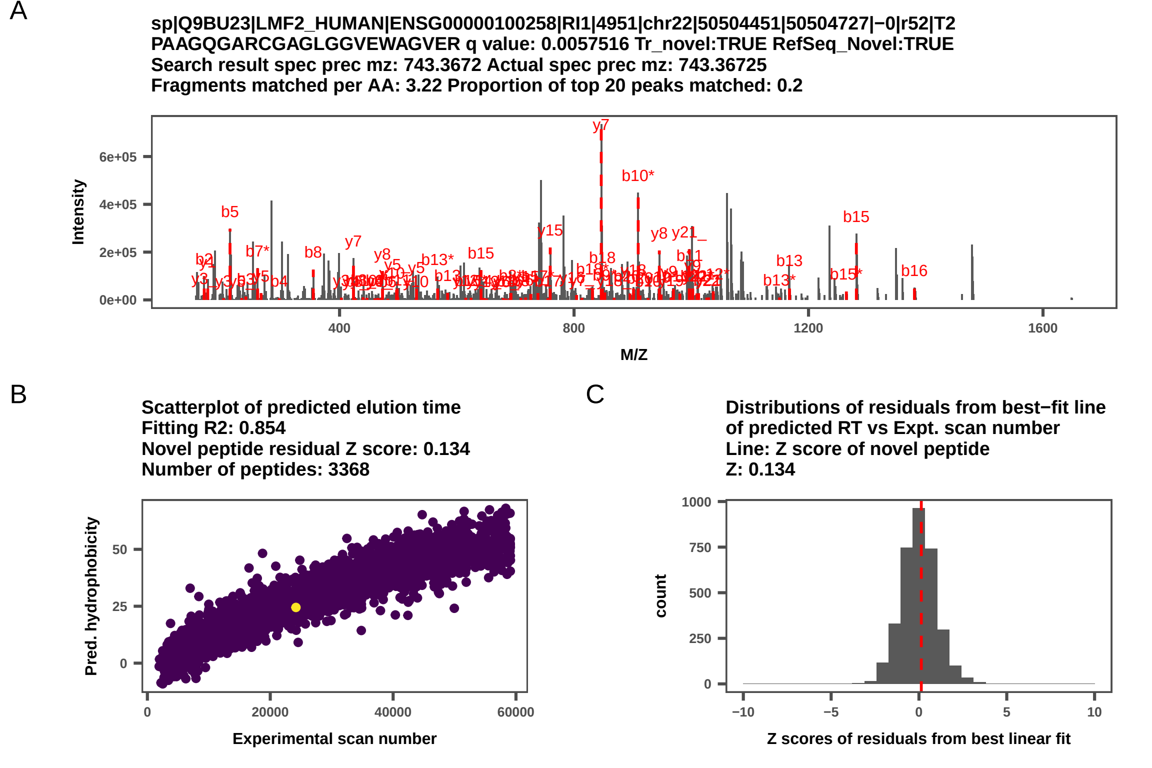
A
sp|Q9BU23|LMF2_HUMAN|ENSG00000100258|RI1|4951|chr22|50504451|50504727|−0|r52|T2
PAAGQGARCGAGLGGVEWAGVER q value: 0.0057516 Tr_novel:TRUE RefSeq_Novel:TRUE
Search result spec prec mz: 743.3672 Actual spec prec mz: 743.36725
Fragments matched per AA: 3.22 Proportion of top 20 peaks matched: 0.2
Intensity
6e+05
4e+05
2e+05
0e+00
y7
b10*
b5
b15
y15
y8 y21_
b7*
b8
y7
y8
b13*
b15
b18
b11
b13
b13*
b15*
b16
400
800
1200
1600
M/Z
B
Scatterplot of predicted elution time
Fitting R2: 0.854
Novel peptide residual Z score: 0.134
Number of peptides: 3368
Pred. hydrophobicity
50
25
0
0
20000
40000
60000
Experimental scan number
C
Distributions of residuals from best−fit line
of predicted RT vs Expt. scan number
Line: Z score of novel peptide
Z: 0.134
count
1000
750
500
250
0
−10
−5
0
5
10
Z scores of residuals from best linear fit
